# Water-Soluble Vitamins Status in Patients Undergoing Maintenance Hemodialysis

**DOI:** 10.3390/nu15020440

**Published:** 2023-01-14

**Authors:** Małgorzata Kaczkan, Sylwia Czaja-Stolc, Małgorzata Szczuko, Arleta Drozd, Przemysław Rutkowski, Alicja Dębska-Ślizień, Sylwia Małgorzewicz

**Affiliations:** 1 Department of Clinical Nutrition, Medical University of Gdansk, 80-211 Gdańsk, Poland; 2 Department of Human Nutrition and Metabolomics, Pomeranian Medical University in Szczecin, 71-460 Szczecin, Poland; 3 Department of Internal and Pediatric Nursing, Medical University of Gdansk, 80-210 Gdańsk, Poland; 4 Department of Nephrology, Transplantology and Internal Medicine, Medical University of Gdansk, 80-952 Gdańsk, Poland

**Keywords:** hemodialysis, water-soluble vitamins, diabetes mellitus

## Abstract

The concentration of water-soluble vitamins (except folic acid and vitamin B_12_) is not routinely measured, which may lead to undiagnosed deficiencies among hemodialysis (HD) patients. The aim of the study was to assess the blood concentration of water-soluble vitamins in HD patients in comparison with healthy subjects and to assess the impact of diabetes mellitus (DM) coexistence on the concentration of these vitamins. The two-center study included 142 HD patients and a control group of 31 healthy subjects. Vitamins concentration was determined using high-performance liquid chromatography (HPLC). Vitamin B_1_, B_6_, and B_12_ levels were significantly lower in the HD group than in the control group (*p* < 0.001). Vitamin B_1_ and B_2_ were negatively correlated with blood urea nitrogen (BUN) levels before HD (R = −0.39, R = −0.38; *p* < 0.05). Vitamin B_3_, B_12_, and C were positively correlated with the albumin concentration (R = 0.26, R = 0.27, R = 0.28; *p* < 0.05). Among diabetic patients, only the concentration of vitamin B_1_ was lower than among non-diabetic patients. The concentration of water-soluble vitamins may be related to the adequacy of dialysis, the time of laboratory determination since the last dialysis, diet, coexistence of other diseases, use of drugs, and dietary supplements in individual patients.

## 1. Introduction

Appropriate diet and nutritional status are important issues in patients with chronic kidney disease (CKD), especially among dialysis patients. Considerable attention is paid to the significant risk of the development of protein-energy wasting (PEW) among hemodialysis (HD) patients, which adversely affects the prognosis of this group of people.

The purpose of hemodialysis is to remove excess water, electrolytes, and uremic toxins that the kidneys are unable to excrete. The HD treatment consists of the exchange of fine-particle substances dissolved in water, between the blood (or more precisely: plasma water) of the patient and the dialysis fluid. The patient’s plasma water is separated from the dialysis fluid by a semi-permeable membrane through which, by diffusion and/or convection, an exchange process takes place. Many elements are important in the effectiveness of the procedure, but the permeability of the dialysis membrane plays a key role, described by KoA (overall mass transfer coefficient). The second mechanism for the transport of solutes across the membrane semi-permeable dialyzer capillary is convection. Because water molecules are very small and freely pass through semi-permeable membranes, hydrostatic pressure (or osmotic) can determine the direction of water passing through a semi-permeable membrane, depending on their value. Larger molecules of solutes, the sizes of which exceed the size of the membrane filtration channels, will be retained and will not pass through it [1,2,3]. HD is associated with a higher loss of vitamins than peritoneal dialysis [4]. In addition, the use of high-flux, high-efficiency membranes in HD has many advantages associated with better removal of uremic toxins, but it is also associated with a greater loss of vitamins [5,6].

HD patients are at risk of developing water-soluble vitamin deficiencies. The reasons for their deficiency are not only losses during HD but also reduced intake with diet, malabsorption in the gastrointestinal tract, and altered vitamin metabolism [6,7]. According to the latest guidelines of the European Society for Clinical Nutrition and Metabolism (ESPEN) on micronutrients, attention has been drawn to the higher risk of deficiency of vitamins B_1_, B_3_, B_6_, and folic acid among patients with CKD, which is associated with the need to increase the intake of these vitamins [8]. In patients with HD, supplementation of water-soluble vitamins is recommended in case of reduced dietary intake [9]. Unfortunately, there is a risk that not every patient has the opportunity to consult a dietitian whose task would be to assess vitamin intake. In addition, patients who are prescribed supplementation may not adhere to these recommendations, which may lead to the development of diseases associated with vitamin deficiency, including skin diseases, immune disorders, and megaloblastic anemia [10].

Folic acid and vitamin B_12_ levels are usually monitored, but the other water-soluble vitamins are not routinely measured in blood. There are also no clear reference values for these vitamins. For some vitamins, the determination of their blood concentration may not be relevant in clinical practice. In some cases, functional tests are performed: for example, transketolase in the case of vitamin B_1_ or glutathione reductase activity in erythrocytes in the case of vitamin B_2_ [11].

Water-soluble vitamins play an important role in the regulation of metabolism and have an antioxidant effect. There are also other substances whose concentration is affected by the use of hemodialysis, such as polyphenols or lactoferrin, which are also antioxidants [12,13]. A study in salt-loaded, high-fat rat models showed that a diet rich in lactoferrin protects the kidneys from inflammation [14]. In another study, lactoferrin suppressed renal fibrosis through the inhibition of apoptosis and the induction of autophagy in a mouse model of folic acid-induced AKI to CKD transition [15].

The study was designed to compare the concentrations of water-soluble vitamins in the blood of HD patients and a healthy control group. Some studies indicate an increased risk of developing complications of diabetes mellitus (DM) among individuals with too little intake and reduced concentration of water-soluble vitamins in the blood [16,17]. For this reason, the study also aimed to check whether the coexistence of DM in HD patients leads to changes in the concentrations of the above-mentioned vitamins in the blood.

## 2. Materials and Methods

### 2.1. Study Design and Population

The study was a two-center, cross-sectional study that was conducted in 2019. The study aimed to assess the blood concentration of water-soluble vitamins in HD patients in comparison with healthy people. The influence of the coexistence of DM on the concentration of these vitamins was also assessed.

**Figure 1:**
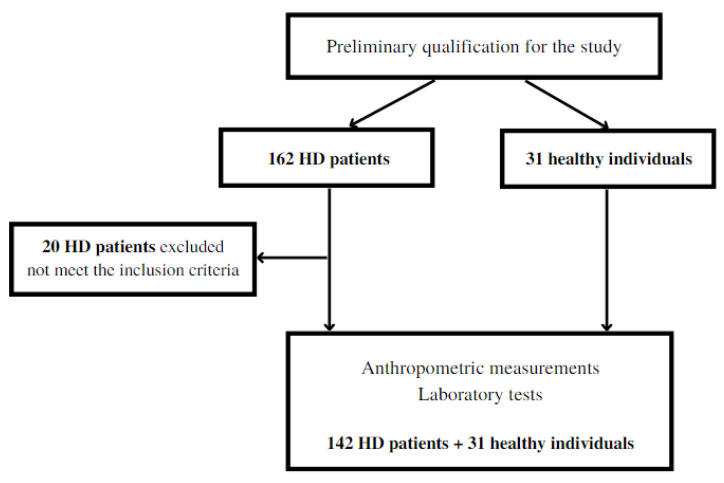
Scheme of the study.

Initially, 162 patients with the 5th stage of CKD were treated with maintenance HD, and 31 healthy volunteers (control group) were qualified for the study. During the in-depth interview, 20 HD patients declared the use of multivitamin supplements. For this reason, they were excluded from the study. The scheme of the study is presented in Figure 1.

The study was approved by the Independent Bioethics Committee of the Medical University of Gdansk (NKBBN/417/2015).

The study involved 142 HD patients in stable clinical status in two dialysis centers located in Poland in the Pomeranian Voivodeship (at the University Clinical Center in Gdańsk and Diaverum in Gdańsk). The inclusion criteria for HD patients were age over 18 years, a minimum 3-month period of HD, and informed and written consent to participate in the study. Patients treated with HD for less than 3 months, with impaired cognitive functions, with acute illnesses, and taking supplementation of water-soluble vitamins in the last 4 weeks were excluded from the study.

All patients enrolled in the study were treated with standard hemodialysis using high-flux dialyzers for an average duration of 4 h (range 3–5 h) three times a week. The blood flow was in the range of 300–350 mL/h. Dialysis adequacy (Kt/V) was 1.65 ± 0.33.

The inclusion criteria for the control group were as follows: age over 18 years and informed and written consent to participate in the study. The exclusion criteria were chronic diseases, infectious diseases, cognitive disorders (such as speech disorders, memory disorders, lack of orientation as to the drugs used, and the occurring disease entities) assessed on the basis of an interview, use of any medications, and supplementation of water-soluble vitamins in the last 4 weeks.

### 2.2. Anthropometric Measurements

Body weight was measured twice, before and after HD. Body weight (kg) and height (meters) were measured using a mechanical column scale with a stadiometer. Body mass index (BMI) was calculated as post-hemodialysis weight divided by the square of height.

### 2.3. Biochemistry

The blood was collected after overnight fasting, before and after a mid-week dialysis session. Biochemical parameters such as blood morphology, blood urea nitrogen (BUN) (before and after HD), serum calcium concentration, phosphorus, sodium, potassium, and albumin were estimated using routine laboratory procedures among HD patients. The normalized rate of protein catabolism (nPCR) was calculated based on the Daugirdas formula [18]. These were tests routinely performed among all patients in the dialysis centers where we qualified patients.

Blood samples for vitamin concentration analysis were taken before HD. In the control group, blood was collected in the morning after overnight fasting. The blood was centrifuged, and the plasma samples were stored at −80 degrees until the assays were performed.

### 2.4. Analysis of Water-Soluble Vitamins with the HPLC Method

Analysis of water-soluble vitamin content was performed using a high-performance liquid chromatography (HPLC) Infinity1260 chromatograph (Agilent Technologies, Waldbronn, Germany). The gradient method with 25 mM HK_2_PO_4_ buffer, pH 7.0—buffer A, and 100% methanol—buffer B was used for the separation of vitamins.

The separation of vitamins was carried out on a Thermo Scientific Hypersil BDS C18 column (2.4 μm, 100 × 4.6 mm; Rockwood TN, USA) at 35 °C. The flow rate of the buffers was 0.9 mL/min, and the injection volume was 10 μL. Individual vitamins in the analyzed samples were identified based on the retention time of the standard peaks. Data analysis was carried out using the ChemStation program (Agilent Technologies, Cheadle, UK), where the amount of each compound was automatically calculated based on the previously obtained standard curves, taking into account the correction for the internal standard (theobromine at a concentration of 100 ng/mL).

For the analysis, 400 μL of plasma was collected, and then the same amount of acetonitrile and 100 μL of internal standard (theobromine at a concentration of 100 ng/mL) was added. All reagents were thoroughly mixed for 2 min and then centrifuged for 15 min at 4000 rpm. The supernatant was collected in new tubes for evaporation of the acetonitrile. The aqueous phase was transferred to solid phase extraction (SPE) columns containing a C-18 silica cartridge (Thermo Scientific, Rockwood TN, USA)—pre-activated with 1 mL methanol and 1 mL ultrapure water. Compounds on the columns were eluted using 85% methanol in 1.5 mL of water. The resulting solution was dried under vacuum and dissolved in 100 μL of buffer A (25 mM HK_2_PO_4_) immediately before HPLC analysis. 

All HPLC grade reagents were purchased from Sigma Aldrich (St. Louis, MO, USA). Buffers were prepared with Millipore grade water (Millipore, Billerica, MA, USA). Amber Eppendorf tubes were used to isolate the vitamins. The vitamin isolation room was darkened to prevent photo-oxidation of the vitamins.

### 2.5. Data Collection

Age, dialysis vintage, cause of renal failure, and DM type 1 and type 2 coexistence were obtained on the basis of individual medical history and access to medical records.

### 2.6. Statistical Analysis

Statistical analysis was carried out using Statistica 13.3 by StatSoft Polska and Microsoft Office Excel 365. Data are presented as the mean ± standard deviation (SD) or median. The variables were assessed for compliance with the normal distribution using histograms and the Shapiro–Wilk test. Differences between the two groups were calculated using Student’s *t*-tests or U Mann–Whitney tests, depending on the distribution. Spearman’s correlation was used for a nonparametric measure of statistical dependence between two variables. Independent associations between variables were assessed by stepwise multiple regression analysis. The level of statistical significance in the study was *p* < 0.05.

## 3. Results

### 3.1. Characteristics of the Study Populations

The baseline characteristics of the HD patients group consisting of 142 people and the control group of 31 people are presented in Table 1. In the group of HD patients, DM coexisted in 38.7% of the patients.

### 3.2. Biochemical Parameters

In HD patients, several laboratory parameters were measured in serum. The results of the biochemical parameters are presented in Table 2. Increased potassium and phosphorus levels were observed. Albumin and calcium levels were decreased.

### 3.3. Blood Concentration of Water-Soluble Vitamins

#### 3.3.1. HD Patients vs. Control Group

**Figure 2:**
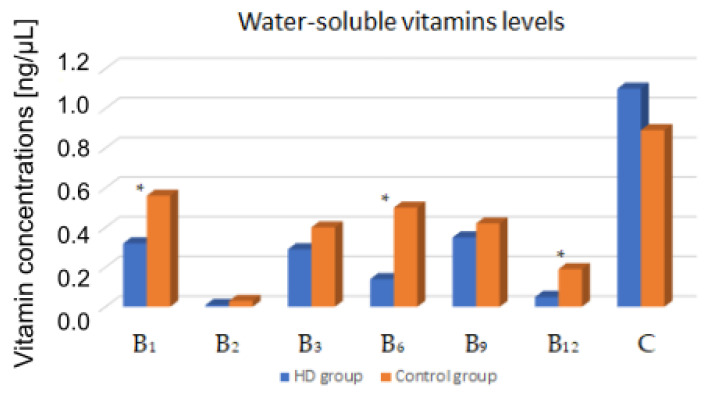
Comparison of blood vitamin levels between HD patients and the control group. *—*p* < 0.05.

The concentrations of water-soluble vitamins were compared between HD patients and a control group. Vitamin B_1_, B_6_, and B_12_ levels were statistically significant lower in studied group (*p* < 0.05). The results are presented in Table 3 and Figure 2.

In the group of HD patients, significant correlations of vitamin concentrations between selected biochemical parameters were observed. Vitamin B_1_ negatively correlated with BUN level before (R = −0.39) and after HD (R = −0.32). Vitamin B_2_ negatively correlated with BUN concentration before HD (R = −0.38). Vitamin B_12_ positively correlated with the concentration of potassium (R = 0.18), albumin (R = 0.27), and nPCR (R = 0.2). Vitamin B_3_ and C were also positively correlated with the albumin concentration (respectively R = 0.26 and R = 0.28).

#### 3.3.2. Multivariate Regression

A multivariate regression model was developed to predict the influence of independent variables related to nPCR, BUN before HD, Kt/V, and albumin on the concentration of water-soluble vitamins in the blood of HD patients.

The analysis showed that Kt/V, nPCR, and BUN before HD may have a significant impact on the concentration of vitamin B_1_ in the blood (the adjusted R^2^ of the model was 0.3; *p* <0.001). Blood folic acid levels in HD patients are affected by Kt/V, nPCR, and BUN before HD, but not albumin levels (the adjusted R^2^ of the model was 0.12; *p* < 0.001). Detailed results of these multiple regressions are presented in Table 4.

#### 3.3.3. HD Patients with and without DM

The concentration of water-soluble vitamins in the blood of patients with and without DM was compared. A significant difference in thiamine concentration was observed. Vitamin B_1_ levels were significantly lower in HD patients with co-existing DM. The results are presented in Table 5.

Patients with DM had a significantly higher BMI than patients without DM (27.7 ± 7.1 vs. 23.5 ± 5.6; *p* <0.05). No differences were observed in the results of biochemical tests.

## 4. Discussion

Water-soluble vitamins are compounds necessary for the proper course of many life processes. Their role consists in regulating metabolic changes, body growth, proper functioning of the nervous system, and red blood cell formation. These vitamins must be part of eaten food or be supplied with dietary supplements because synthesis in the body is impossible (with the exception of some synthesis of niacin, and folic acid). B vitamins and vitamin C are present in vegetables, fruits, cereal products, legumes, dairy products, meat, and eggs. The deficiency of these vitamins can be present in alcohol use disorder, malabsorption syndromes, malnutrition, and in people using an incorrectly balanced vegan diet [19,20,21].

This two-center cross-sectional study showed that vitamin B_1_, B_6_, and B_12_ levels were significantly lower in HD patients than in the control group. We did not find a similar study in the sources available to us. Most of the studies concern the loss of water-soluble vitamins with the dialysate or patients supplementing the mentioned vitamins. In research by Hong et al., dialysis patients had higher concentrations of vitamin B_6_ and B_12_ compared to the control group, but patients were supplemented daily with 2 mg of pyridoxine and 2 mg of cyanocobalamin [22].

The first article about vitamins and minerals lost during HD was published in the 1980s [23]. Another study found important losses of water-soluble vitamins (B_1_, B_6_, B_9_, and C) during a standard 4 h hemodiafiltration session [24]. Schwotzer et al. found that the levels of most vitamins are above the normal range in patients on hemodiafiltration receiving a classic dose of vitamin supplements, vitamin C excepted [25].

In our study, we observed a correlation between the concentrations of individual vitamins and biochemical parameters among HD patients. Vitamin C, B_3_, and B_12_ levels were positively correlated with the albumin concentration, indicating that people with better nutritional status also have higher concentrations of certain vitamins. Vitamin B_12_ was also positively correlated with nPCR, which is related to the value of daily protein intake. In the multiple regression model, the concentration of vitamin B_1_ and folic acid was dependent on the adequacy of dialysis, nPCR, and BUN concentration before HD.

Vitamin B_1_ is necessary for the course of many metabolic processes. Its derivatives are essential cofactors of important enzymes responsible for the metabolism of amino acids and carbohydrates. Good sources of vitamin B_1_ in the diet are wholegrain cereal products, meat, legumes, seeds, and nuts. These products are also a good source of phosphate. The recommended low phosphate diet among HD patients may contribute to a reduction in vitamin B_1_ intake [26]. Thiamine stores are low and the use of an improperly balanced diet for several weeks can lead to its deficiency, especially in the case of HD patients, because the hemodialysis procedure causes the loss of this vitamin. The loss of vitamin B_1_ during dialysis depends on the duration of the procedure and the type of hemodialysis used [6,24]. Jankowska et al. measured that thiamine diphosphate (TDP), a bioactive compound of vitamin B_1_, is substantially lost during the HD procedure, and the amount of its loss is associated with body weight. It is not influenced by vitamin B_1_ dietary intake and standard supplementation dose [27].

In our study, the HD group had significantly lower vitamin B_1_ levels than the control group (0.32 ± 0.34 ng/μL vs. 0.56 ± 0.38 ng/μL). In addition, we observed that thiamine concentrations were significantly lower among diabetic patients. Only the concentration of vitamin B_1_ was significantly different among HD diabetic patients compared to patients without DM. According to the sources available to us, this is the first publication comparing the concentration of water-soluble vitamins among HD patients with and without DM. Wernicke’s encephalopathy associated with vitamin B_1_ deficiency is currently diagnosed very rarely. Most often, its occurrence is associated with people abusing alcohol. However, in the available literature, there are case reports of Wernicke’s encephalopathy in HD patients for diabetic nephropathy [28,29]. The co-occurrence of DM has not been shown to contribute to the presence of these neurological disorders, but it has been observed that thiamine metabolism is altered in DM. The presence of hyperglycemia stimulates glycolysis and the formation of reactive oxygen species. The pentose phosphate pathway (PPP) is an alternative mode of glucose metabolism when glycolysis is overloaded. Vitamin B_1_ is a cofactor of enzymes involved in PPP; therefore, its use increases among patients with DM [30,31,32]. Vitamin B_1_ deficiency among DM patients may contribute to the development of diabetic nephropathy [16,33,34].

Riboflavin plays a key role in energy metabolism. The deficiency of vitamin B_2_ is caused by its insufficient supply in the diet and impaired absorption [2]. In our study, we did not find differences between the levels of this vitamin in the blood of the study and the control group, even if they did not receive any systematic supplementation in two centers. In our previous study from 2016, we observed that 48% of HD patients had inadequate dietary vitamin B_2_ intake [35], and in a study by other authors, the intake was too low in about 25% of patients [36]. Bevier et al. observed during the dialysis procedure the loss of vitamin B_2_ is 13% [24]. For this reason, it is necessary to watch the intake of this vitamin along with the diet.

Vitamin B_3_ is crucial in the synthesis of carbohydrates, proteins, and fatty acids and therefore plays a key role in energy metabolism. The deficiency of niacin results in a photosensitive pigmented dermatitis called pellagra. Ramirez et al. found that niacin levels do not change pre- and post a single hemodialysis treatment [37]. In our study, we did not observe differences in the concentration of vitamin B_3_ between the study group and the control group. Supplementation with niacin derivatives may reduce blood phosphate levels in CKD patients by reducing absorption in the gastrointestinal tract as a result of inhibiting sodium-dependent phosphate co-transport [38].

Vitamin B_6_ is necessary for the proper metabolism of proteins and the synthesis of nucleic acids as well as transformations of fats, carbohydrates, and fat-soluble vitamins. It takes part in the production of antibodies and red blood cells [39]. Vitamin B_6_ deficiencies are most often correlated with deficiencies of other B vitamins and often affect the elderly and those on HD as well as people with homocysteinemia [9]. In our previous study, we observed that 40% of HD patients had low dietary vitamin B_6_ intake [34]. Bevier et al. found that vitamin B_6_ concentrations decreased by 25.4% after one dialysis session [24]. In our study, the HD group had significantly lower levels of this vitamin than the control group (0.14 ± 0.16 ng/μL vs. 0.5 ± 0.26 ng/μL). In a study comparing vitamin B levels among people with and without DM (non-dialyzed), it was observed that vitamin B_6_ levels were significantly lower among people with DM [40]. In our study, no such differences were observed among HD patients with and without DM. The National Kidney Foundation’s Kidney Disease Outcomes Quality Initiative (NKF/KDOQI) highlighted that a daily supplement of 10 mg of pyridoxine has been recommended in adult dialysis patients [9].

Folic acid is involved in the transformation of amino acids and the synthesis of nucleic acids, and it is essential for proper growth and development and for the production of red blood cells. It affects the proper function of the nervous system, digestive tract, and the course of homocysteine metabolism. Deficiencies of this vitamin occur in people using an unbalanced diet or having increased demand. Green leafy vegetables, whole grains, yeast, and liver are particularly rich in folate. Intestinal bacteria also synthesize folate. Bevier et al. and Schwotzer et al. described vitamin B_9′_s important losses per season [24,25]. Coveney et al. found that extended hours of dialysis did not have an impact on serum levels of folate in 52 HD patients, 38% of whom were taking a multivitamin [7].

Vitamin B_12_, involved in 1-carbon metabolism, is an essential nutrient for nucleotide and amino acid biosynthesis. Deficiency is a common cause of hyperhomocysteinemia and a frequent feature of patients with CKD. Homocysteine metabolism forms an important component of the vitamin B_12_ metabolic pathway. Impairment in vitamin B_12_ metabolism is considered a nontraditional risk factor for poor outcomes associated with CKD. Cardiovascular events are the leading causes of death in these patient populations; thus, the interest in non-traditional risk factors such as hyperhomocysteinemia, folic acid, and vitamin B_12_ metabolism is growing [41,42]. Diet is an important determinant of vitamin B_12_ status. The main source of cobalamin is from dietary intake of animal and dairy products. In our study, the HD group had significantly lower vitamin B_12_ levels than the control group 0.05 ± 0.04 ng/μL vs. 0.19 ± 0.17 ng/μL. Cobalamin losses are probably low in hemodiafiltration. Bevier et al. showed no adsorption by the dialyzer membrane [24]. Crews et al. observed that poor dietary habits are common among the urban poor and are strongly associated with their greater prevalence of CKD [43].

Vitamin C in the human body has many functions. It is a part of a number of enzymes and coenzymes; it participates in oxidation and reduction reactions. It prevents the formation of free radicals as well as the aging process. Deficiencies are observed especially in patients with significantly reduced consumption of vegetables and fruits and low potassium diet [44]. Morena et al. reported diffusive losses of vitamin C during hemodiafiltration which cause a loss of 66 mg per session [45]. Bevier et al. reported that 40% of patients had pre-dialysis plasma values below normal, despite a systematic oral supplementation implemented [24]. In our study, we did not find differences between blood vitamin C levels in the study and the control group.

Our study has limitations. The size and age of the study group and the control group are different. We had difficulties in matching the appropriate control group because the exclusion criteria for participation in the study were the presence of chronic diseases, the use of any medications, and dietary supplements. People on hemodialysis are usually older people, so we had no opportunity to match a control group of the same age because, in this age group, patients usually have cardiovascular disease and take many medications. We assumed that our study would include HD patients not taking water-soluble vitamin supplements for 4 weeks. However, it would be interesting to compare whether the use of supplementation significantly affects the concentration of these vitamins in the blood. We also did not assess the patients’ nutrient intake, which would allow us to extend the study to include the effect of the diet on the concentration of water-soluble vitamins.

## 5. Conclusions

Vitamin B1, B6, and B12 levels are lower in HD patients than in controls. This may be related to the adequacy of dialysis, losses of these vitamins during procedures, and diet. Vitamin B1 concentration was significantly lower among diabetic patients, which may be related to the increased demand for this vitamin in this group of patients.

## 6. Clinical Implication

Our study especially showed vitamin B_1_, B_6_, and B_12_ deficiency. The DOPPS study showed a 16% reduction in the relative risk of death in HD patients taking water-soluble vitamins [9]. Therefore, patients should be encouraged to take water-soluble vitamin supplements regularly, as well as recommended supplements suitable for dialysis patients. Specific guidelines on the dosages and forms of dietary supplements that are most appropriate for these patients would also be useful.

## Figures and Tables

**Table 1:** Characteristics of the study populations.

Parameters	HD Patients *n* = 142	Control Group *n* = 31
Female/Man	57/85	16/15
Female/Man (%)	40.1/59.9	51.6/48.4
Age (years) range	64 ± 15.2 (66) 21–97	41.1 ± 10 (38) 30–72
BMI (kg/m^2^)	25.1 ± 6.5 (24.6)	26.6 ± 4.1
Dialysis vintage (months)	51.6 ± 54.2 (33)	-
Kt/V	1.65 ± 0.33 (1.67)	-
nPCR (g/kg/day)	1.08 ± 0.24	-
Common causes of CKD		
Unknown nephropathy (%)	19.1	
Diabetic nephropathy (%)	15.6	
Chronic glomerulonephritis (%)	14.5	
Hypertensive nephropathy (%)	5.2	
Chronic pyelonephritis (%)	5.2	
Polycystic kidney disease (%)	4.1	

Data are presented as mean ± SD and (median) if the distribution is not normal. BMI—body mass index; Kt/V—dialysis adequacy; nPCR—normalized protein catabolic rate; CKD—chronic kidney disease.

**Table 2:** Biochemical parameters in the HD group.

Parameters	HD Patients *n* = 142	References Values
Hemoglobin (g/dL)	10.7 ± 1.3 (10.7)	9.5–12.5
Albumin (g/L)	35.6 ± 3.9 (36)	38–48
BUN before HD (mg/dL)	57.4 ± 12.6	8.4–25.7
BUN after HD (mg/dL)	14.6 ± 4.5 (13.5)	8.4–25.7
Sodium (mmol/L)	137.1 ± 2.9	136–145
Potassium (mmol/L)	5.3 ± 0.6	3.5–5.1
Calcium (mg/dL)	8.5 ± 0.9 (8.5)	8.9–10
Phosphorus (mg/dL)	5.1 ± 1.3	2.3–4.7

Data are presented as mean ± SD and (median) if the distribution is not normal. BUN—blood urea nitrogen; HD—hemodialysis.

**Table 3:** Comparison of blood vitamin levels between HD patients and the control group.

Vitamin	HD Patients *n* = 142	Control Group *n* = 31	*p*
B_1_—thiamine (ng/μL)	0.32 ± 0.34 (0.15)	0.56 ± 0.38	<0.001
B_2_—riboflavin (ng/μL)	0.009 ± 0.007 (0.006)	0.03 ± 0.03 (0.008)	0.17
B_3_—niacin (ng/μL)	0.29 ± 0.21 (0.32)	0.4 ± 0.38 (0.54)	0.12
B_6_—pyridoxine (ng/μL)	0.14 ± 0.16 (0.08)	0.5 ± 0.26	<0.001
B_9_—folic acid (ng/μL)	0.35 ± 0.34 (0.19)	0.42 ± 0.28	0.13
B_12_—cobalamin (ng/μL)	0.05 ± 0.04 (0.03)	0.19 ± 0.17 (0.13)	<0.001
C—ascorbic acid (ng/μL)	1.1 ± 0.79 (0.88)	0.89 ± 0.22 (0.93)	0.69

Data are presented as mean ± SD and (median) if the distribution is not normal.

**Table 4:** Influence of adequacy of dialysis, nPCR, BUN before HD, and albumin level on the concentration of vitamins B_1_ and B_9_ in the blood of HD patients.

Regression Model	B	Standard Error	Beta	*p*-Value
Vitamin B_1_ concentration				
Constant	−1.3	0.51		0.02
Kt/V	1.91	0.49	1.84	<0.001
nPCR	−5.25	1.21	−3.96	<0.001
BUN before HD	0.07	0.02	3.26	<0.001
Vitamin B_9_ concentration				
Constant	−2.15	1.04		0.049
Kt/V	1.9	0.77	1.41	0.02
nPCR	−4.93	1.91	−2.78	0.02
BUN before HD	0.06	0.03	2.39	0.02
Albumin	0.03	0.02	0.25	0.16

Kt/V—dialysis adequacy; nPCR—normalized protein catabolic rate; BUN—blood urea nitrogen; HD—hemodialysis.

**Table 5:** Comparison of blood vitamin levels between HD patients with and without DM.

Vitamin	HD Patients with DM *n* = 55	HD Patients without DM *n* = 87	*p*
B_1_—thiamine (ng/μL)	0.24 ± 0.28 (0.09)	0.36 ± 0.37 (0.28)	0.04
B_2_—riboflavin (ng/μL)	0.009 ± 0.008 (0.006)	0.009 ± 0.006 (0.007)	0.59
B_3_—niacin (ng/μL)	0.28 ± 0.22 (0.31)	0.29 ± 0.21 (0.35)	0.7
B_6_—pyridoxine (ng/μL)	0.15 ± 0.15 (0.09)	0.14 ± 0.17 (0.07)	0.44
B_9_—folic acid (ng/μL)	0.31 ± 0.34 (0.12)	0.39 ± 0.34 (0.28)	0.16
B_12_—cobalamin (ng/μL)	0.04 ± 0.02 (0.03)	0.05 ± 0.04 (0.04)	0.72
C—ascorbic acid (ng/μL)	1.1 ± 0.91 (0.83)	1.1 ± 0.71 (0.98)	0.41

Data are presented as mean ± SD and (median) if the distribution is not normal.

## Data Availability

Not applicable.
